# Current and Future Imaging Methods for Evaluating Response to Immunotherapy in Neuro-Oncology

**DOI:** 10.7150/thno.34415

**Published:** 2019-07-09

**Authors:** Benjamin B. Kasten, Neha Udayakumar, Jianmei W. Leavenworth, Anna M. Wu, Suzanne E. Lapi, Jonathan E. McConathy, Anna G. Sorace, Asim K. Bag, James M. Markert, Jason M. Warram

**Affiliations:** 1 Department of Neurosurgery, University of Alabama at Birmingham, Birmingham, AL, United States.; 2 School of Medicine, University of Alabama at Birmingham, Birmingham, AL, United States.; 3 Crump Institute for Molecular Imaging, Department of Molecular and Medical Pharmacology, David Geffen School of Medicine at University of California Los Angeles, Los Angeles, CA, United States.; 4 Department of Radiology, University of Alabama at Birmingham, Birmingham, AL, United States.; 5 Department of Diagnostic Imaging, St. Jude Children's Research Hospital, Memphis, TN, United States.; 6 Department of Otolaryngology, University of Alabama at Birmingham, Birmingham, AL, United States.

**Keywords:** neuro-oncology, immunotherapy, imaging, response, MRI, PET

## Abstract

Imaging plays a central role in evaluating responses to therapy in neuro-oncology patients. The advancing clinical use of immunotherapies has demonstrated that treatment-related inflammatory responses mimic tumor growth via conventional imaging, thus spurring the development of new imaging approaches to adequately distinguish between pseudoprogression and progressive disease. To this end, an increasing number of advanced imaging techniques are being evaluated in preclinical and clinical studies. These novel molecular imaging approaches will serve to complement conventional response assessments during immunotherapy. The goal of these techniques is to provide definitive metrics of tumor response at earlier time points to inform treatment decisions, which has the potential to improve patient outcomes. This review summarizes the available immunotherapy regimens, clinical response criteria, current state-of-the-art imaging approaches, and groundbreaking strategies for future implementation to evaluate the anti-tumor and immune responses to immunotherapy in neuro-oncology applications.

## I. Introduction

Central nervous system (CNS) malignancies are associated with significant morbidity and mortality. In adults, the extremely aggressive glioblastoma (GBM) has the highest incidence of all gliomas and causes more deaths than all other primary CNS malignancies combined. GBM is associated with a median overall survival of less than two years despite surgery and chemo-radiotherapy 1. Patients with secondary CNS metastases of lung cancer, melanoma, or breast cancer, among others, also suffer poor outcomes due to the resistance of these lesions to available treatments 2. Thus, a growing number of novel therapeutic strategies, such as immunotherapy, are being investigated in an attempt to improve the prognosis for neuro-oncology patients 1, 3-7.

Cancer immunotherapy strategies aim to stimulate innate or adaptive immune responses against malignant cells. During the last decade, immunotherapy regimens have made remarkable clinical progress in cancer patients, particularly those with recalcitrant solid tumors, by overcoming immune-suppressive signals present in the tumor microenvironment 8-10. While the brain was historically viewed as an immune-privileged organ due to the blood-brain barrier (BBB), this paradigm has been changed as a growing number of studies have demonstrated the cross-talk between systemic and CNS-resident immune cells. Immunotherapy strategies that had traditionally been considered irrelevant for CNS diseases are now being pursued to determine their potential to improve survival in patients who are refractory to the traditional paradigm of treatment (maximal safe resection and adjuvant chemo-radiotherapy) 11, 12.

Non-invasive, longitudinal imaging is central to measuring the progression of disease and the efficacy of treatment in neuro-oncology. Historically, response criteria based on conventional imaging through computed tomography (CT) or magnetic resonance imaging (MRI) techniques were used to monitor changes in tumor morphology and responses to tumoricidal chemo-radiotherapy regimens, where shrinkage corresponded with malignant cell death and therapeutic response, while enlargement or appearance of new foci corresponded to therapeutic failure and progressive disease. Studies over the last two decades with emerging biologic or immunotherapy regimens, which are predominantly tumoristatic, have shown the failure of traditional morphologic response criteria and conventional imaging techniques to predict therapeutic outcomes. Inflammatory responses and related effects from immunotherapy are often indistinguishable from progressive disease during conventional imaging, particularly at early stages of treatment 13, 14. This phenomenon of early therapy apparent tumor progression on imaging followed later by tumor regression and clinical improvement has been termed pseudoprogression 15. Distinguishing between pseudoprogression and progressive disease is critical for clinicians to manage patient care most effectively, as either prolonged use of an ineffective treatment or premature cessation of a currently effective treatment could negatively impact patient outcome.

An expanding effort is being mounted to address these challenges through modified response criteria and advanced imaging techniques in research and clinical settings 16-18. The purpose of this review is to summarize the current state and future directions of non-invasive imaging for monitoring immunotherapy responses in neuro-oncology studies. The sections below will briefly discuss relevant response criteria and advanced imaging techniques that have been used with immunotherapy strategies, as well as primary literature that incorporates non-invasive imaging and immunotherapy in neuro-oncology studies.

## II. Review of response criteria and imaging techniques relevant to immunotherapy in neuro-oncology

### A. Criteria used to evaluate response to therapy in neuro-oncology

Non-invasive imaging in neuro-oncology aims to define widely applicable clinical response criteria for evaluating disease progression and comparing response outcomes across studies. However, distinguishing between tumor response, pseudoprogression, and progressive disease is a significant challenge in neuro-oncology patients treated with immunotherapy. Pseudoresponse is an alternative imaging pattern where the malignancy falsely appears to have responded to a particular therapy regimen 19, 20. Pseudoprogression has been commonly reported during immunotherapy in case reports or exploratory neuro-oncology studies 13, 14, while pseudoresponse during immunotherapy has not been widely documented in neuro-oncology patients. Relatively high rates of pseudoprogression (36%) have been reported in a systematic meta-analysis of neuro-oncology studies incorporating patients treated with traditional chemo-radiotherapy 21, although objective rates of pseudoprogression associated with immunotherapy in neuro-oncology have not been systematically reported. Considering the increased rates of pseudoprogression observed during immunotherapy relative to chemo-radiotherapy in non-CNS malignancies 22, similar trends are likely to emerge from ongoing and future immunotherapy studies in neuro-oncology. The variable patterns of response to established and emerging therapy regimens have impacted the clinical criteria used to assess neuro-oncology patients. A brief overview of established and emerging neuro- oncology response criteria published within the last two decades is presented here. Several recent reviews are available for more detailed discussion of these criteria 23-26.

The Macdonald criteria, proposed in 1990 27, measured GBM tumor burden by the maximal cross-sectional area of enhancing lesions identified in CT scans or MRI. Progressive disease was defined by 25% increase (or more) in enhancing lesion area, appearance of new lesions, or clinical deterioration. Additional metrics were developed to define stable disease, partial response, and complete response. These criteria enabled comparison of progression free survival between clinical trials. Limitations of the criteria included lack of assessment of diffuse tumor burden, low grade tumors that lacked contrast enhancement, and pseudoresponse patterns that became evident during novel therapeutic development, such as anti-angiogenic antibody therapy studies 28.

The response assessment in neuro-oncology (RANO) criteria were introduced in 2010 29. One rationale for the RANO criteria was to address pseudoprogression observed during MRI in patients treated with radiation and temozolomide chemotherapy 30. RANO criteria departed from Macdonald criteria by including non-contrast-enhancing, fluid-attenuated inversion recovery (FLAIR) MRI measurements and spatial-temporal factors to distinguish between pseudoprogression and progressive disease. Progressive disease was not definitively declared if new lesions or increasing enhancement was observed in irradiated target areas of the brain within 12 weeks of radiation therapy. RANO criteria became available near the start of the rapid expansion in clinical immunotherapy. Resultantly, these criteria have been used more than other criteria for recent clinical immunotherapy trials in neuro-oncology. The RANO group considered the impact of available advanced imaging techniques in neuro-oncology and decided that insufficient data was available at that time to warrant inclusion of these techniques in routine patient assessment. However, the group envisioned that advanced imaging could later be incorporated into response criteria pending outcomes of future clinical trials that utilize advanced imaging techniques.

There are currently hundreds of concurrent clinical trials investigating various immunotherapy strategies in neuro-oncology. These studies have shown ongoing challenges to monitor therapeutic efficacy due to variations in response patterns observed during immunotherapy as opposed to traditional chemo-radiotherapy. The immunotherapy RANO (iRANO) criteria were published in 2015 to guide response assessment specifically in neuro-oncology patients treated with immunotherapy 31, following iterations of other immune response evaluation criteria developed in general for solid tumors (i.e. irRC, irRECIST, iRECIST) 32. The iRANO guidelines were drafted prior to availability of data from large phase III immunotherapy trials in neuro-oncology patients and are thus intended to accommodate emerging data from current and future clinical studies flexibly. Considering the likelihood of pseudoprogression and the timeframe of responses observed in patients with solid tumors treated with checkpoint blockade immunotherapy 22, 31, 33, iRANO criteria do not declare progressive disease if new brain lesions appear within 6 months of starting immunotherapy. Treatment could be continued in these cases for another 3 months or until follow-up radiographic imaging confirmed true progressive disease. The RANO group recommends that extended time frames, allowing for new lesions to appear subsequent to follow-up imaging, are necessary to confirm disease progression and should be tested and adjusted as evidence from ongoing and prospective immunotherapy trials emerge.

The RANO and iRANO criteria were developed primarily for patients with GBM, with the understanding that separate criteria would be required to most effectively define responses to therapy in patients with low grade glioma or with metastases from non-CNS malignancies. RANO characterizes low grade glioma tumor burden by FLAIR signal area rather than by contrast enhancing area, but is otherwise homologous to high grade glioma criteria 34. Criteria for evaluating brain metastases were proposed from the RANO brain metastases (RANO-BM) working group in 2015 35. These criteria are similar to response evaluation criteria in solid tumors (RECIST) 1.1 in that contrast enhancing lesions are measured based on unidimensional diameter during conventional radiographic imaging. Like the iRANO criteria, the appearance of new lesions during immunotherapy is not sufficient alone to define progressive disease unless follow up imaging confirms progression. One study has prospectively evaluated response to anti-PD-1 immunotherapy with pembrolizumab in 36 patients with melanoma or non-small cell lung cancer brain metastases 36. High concordance between four criteria (RECIST1.1, mRECIST, RANO-HGG, and RANO-BM) was observed, although up to 19 patients were not evaluable by RANO-HGG due to lesions that were less than the required size (10 mm) for RANO-HGG assessment. iRANO criteria was not used in the study as those guidelines were published after completion of the study period 36.

### B. Overview of conventional and advanced imaging techniques used in neuro-oncology

**Figure 1:**
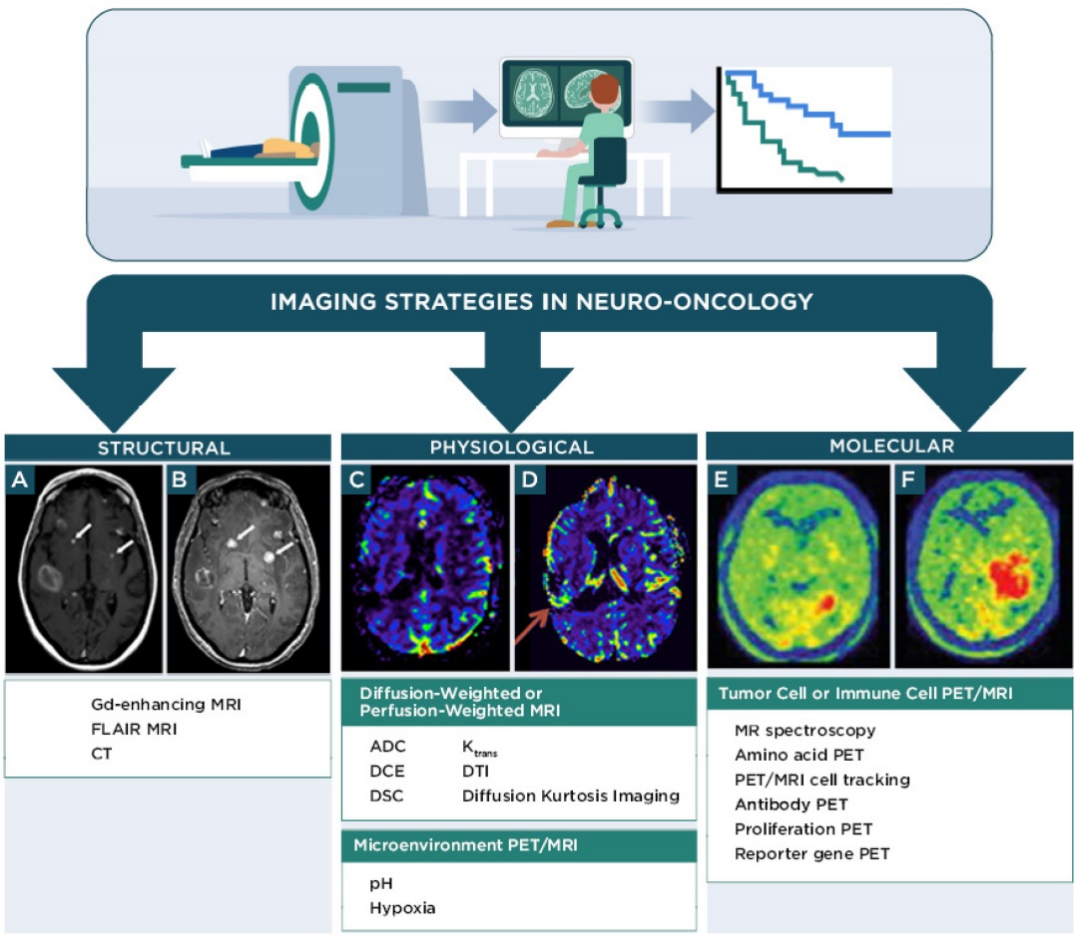
** Overview of imaging techniques used to predict response to immunotherapy in neuro-oncology research. A, B:** gadolinium-enhanced *T_1_* MRI images in a patient with melanoma brain metastases before (A) and 11 days after (B) treatment with pembrolizumab checkpoint immunotherapy. Arrows indicate resected lesions, which showed inflammatory cell responses attributed to treatment effects rather than disease progression. (Adapted with permission from 46 copyright 2015 American Association for Cancer Research) **C,D:** Perfusion weighted MRI images of relative cerebral blood volume in a patient with relapsed malignant glioma at 1.5 months (C) and 10.5 months (D) after treatment with dendritic cell vaccine immunotherapy. The arrow in D indicates increased relative cerebral blood volume in the region of the tumor attributed to progressive disease at the latter time point. (Adapted with permission from 47 copyright 2010 Springer Nature) **E,F:** [^11^C]MET amino acid PET images in a patient with recurrent GBM before (E) and 12 weeks after (F) treatment with WT1 peptide vaccine immunotherapy. Increased PET signal (red) is attributed to disease progression. (Adapted with permission from 48 copyright 2012 American Association of Neurological Surgeons) DCE, dynamic contrast-enhanced MRI; *K*_trans_, rate of transfer from plasma to extravascular space in perfusion MRI; DSC, dynamic susceptibility-weighted contrast-enhanced MRI; ADC, apparent diffusion coefficient in diffusion MRI; DTI, diffusion tensor imaging MRI; DKI, diffusion kurtosis imaging MRI.

Non-invasive imaging techniques for neuro-oncology can be grouped into the following categories: 1) structural imaging through conventional CT or MRI, 2) physiological imaging through advanced MRI, positron emission tomography (PET), or single photon emission computed tomography (SPECT) and 3) molecular imaging through advanced MRI, PET, or SPECT (Figure 1, Table 1). Advanced MRI and PET imaging are increasingly being harnessed in neuro-oncology studies, particularly for defining the extent and progression of disease, identifying individuals who will likely benefit from a specific type of therapy, and monitoring response to therapy 37. Relative to conventional or advanced MRI techniques, PET imaging has much higher sensitivity and is readily amenable to volumetric analyses. Most clinical PET scanners have a limited spatial resolution of 3-5 mm and are often paired with CT or MRI for anatomical reference. The RANO group has recommended including PET imaging (2-deoxy-2[^18^F]fluoro-D-glucose [FDG] or radiolabeled amino acids) in clinical care and in trials that monitor outcomes to therapy, with the expectation that PET parameters could be incorporated into future response criteria in neuro-oncology 38, 39. The following sections briefly discuss major classes of advanced MRI and PET imaging strategies employed in clinical neuro-oncology studies and that are relevant for monitoring response to immunotherapy. Additional information for these and other imaging techniques is available from recent reviews 40-43.

The goal of non-invasive imaging in the context of immunotherapy response assessment is to define diagnostic or prognostic metrics of tumor response, particularly at early time points after initial diagnosis or starting a therapeutic regimen. Imaging can also be used to stratify patients for specific targeted therapies (e.g., checkpoint immunotherapy) based on the expression of biomarkers that correspond to the therapeutic regimen. While biopsy or surgical tissue samples can provide information for tumor progression or biomarker expression, tissue sample analyses are subject to sampling error, may not be representative of spatial-temporal tumor heterogeneity, and are not always available from essential CNS regions or serial time points. Conversely, non-invasive advanced imaging strategies allow global, serial assessment to predict response to treatment or identify biomarker expression 14, 37, 41, 44, 45. As indicated above for the RANO and iRANO criteria, neuro-oncology patients treated with immunotherapy have a significant time window (6-9 months) where a therapy can be continued before conventional radiographic imaging establishes the presence or absence of progressive disease 31. Considering the poor prognosis associated with GBM or CNS metastases, particularly at recurrence, patients that experience significant disease progression during that time window have extremely limited treatment options. Novel imaging strategies are being sought to correlate therapeutic response, disease progression, or patient survival to identifiable imaging metrics at the earliest time point possible, prior to patient deterioration or confirmed disease progression through conventional radiographic parameters. By identifying and implementing such imaging parameters in clinical research, patients that are identified as responders despite initial pseudoprogression can be kept on an effective therapy until remission is established, while patients that are not responding can be switched to another therapeutic regimen before significant progression has occurred. These outcomes would benefit patients by maximizing the window of opportunity to find an effective therapy and reducing unnecessary adverse effects associated with an ineffective therapy.

#### 1. Structural imaging techniques

Conventional CT and MRI techniques that evaluate morphologic (structural, Figure 1) features remain the most common imaging modalities in neuro-oncology. Contrast enhancement on *T_1_* MRI and/or extent of *T_2_* hyperintense areas observed on FLAIR MRI sequences are currently standard parameters used to monitor tumor progression in neuro-oncology. However, enlargement of MRI contrast enhancing components and/or enlargement of FLAIR hyperintense areas can be seen both in tumor progression and in inflammatory responses associated with immunotherapy (Figure 1A, B), and are indistinguishable in most of the circumstances. Because gliomas are invasive on a cellular level, the borders of the tumor cannot be visualized on conventional MRI. The changes seen on more sensitive conventional MRI sequences (e.g., FLAIR) taper off until the concentration of tumor cells within normal parenchyma is too low to affect the signals. Additionally, changes that occur as a result of treatment effect, such as pseudoprogression or inflammation following chemotherapy, radiotherapy or immunotherapy, can be impossible to distinguish from tumor progression on current imaging sequences. Attempts have been made to find more reliable prognostic parameters from rates of change in serial *T_1_* or *T_2_* MRI signals. Kinetic modeling of GBM proliferation and invasion as measured by MRI has been proposed as predictive parameters for response to conventional therapy 77, 78. Future studies would be needed to determine if these analyses could be extended to response assessment during immunotherapy, since it is not yet known if favorable responses to immunotherapy correlate to kinetic changes in these MRI parameters. Treatment response assessment map MRI techniques based on the pattern of contrast accumulation and washout using early (within 5 min) and delayed (after 1 h) contrast-enhanced MRI has been developed to correlate to treatment outcomes in neuro-oncology. This technique has been attempted in monitoring early response to a dendritic cell vaccine immunotherapy 49, while its utility to distinguish pseudoprogression from progressive disease has yet to be tested.

#### 2. Physiological imaging techniques

Physiological imaging (Figure 1) assesses fluid diffusion, perfusion, or microenvironmental conditions differing between malignant and healthy tissues. Several of these advanced imaging techniques, including MRI diffusion weighted imaging (DWI) and perfusion weighted imaging (PWI) parameters, are in routine clinical practice. Though physiological imaging techniques have been used in immunotherapy clinical trials in neuro-oncology patients, it is still unclear from the limited data available which parameters are most useful for predicting patterns of response.

##### a. DWI MRI techniques

DWI MRI parameters measure the diffusion of water molecules in a tissue of interest. The apparent diffusion coefficient (ADC) metric is used in clinical practice and in clinical trials to evaluate response. This parameter is an indication of water diffusivity, which is inversely proportional to cellularity 40, 55, 79, and has been used to monitor response to therapy in several immunotherapy trials. Increase in cellular density is expected in both tumor progression and during the initial stages of immunotherapy, making interpretation of diffusion MRI results challenging. As response to therapy continues, however, cellularity decreases due to elimination of tumor cells and diffusion parameters readily distinguish between immunotherapy response and progressive disease.

i. Diffusion tensor imaging (DTI) MRI adds directionality to DWI and has been used as an anatomic tool to map white matter tracts (fractional anisotropy parameters). DTI is also useful in functional MRI assessment 49. A preclinical study has used DTI to image slow-growing, diffuse GBM models in mice 80, though this study did not look at tumor response during immunotherapy. DTI fractional anisotropy analyses showed correlations between patient outcomes and CD3^+^ cell densities at the interface between the brain and secondary brain metastases in a cohort of 26 patients, suggesting that DTI could be a surrogate marker for immune cell response 50. Regions showing low fractional anisotropy beyond the tumor edge in these patients had high densities of CD3^+^ cells, suggesting that immune cell localization can be detected by low fractional anisotropy values in peritumoral regions. While the effects of immunotherapy were not investigated, these findings support future studies to determine if similar fractional anisotropy patterns occur during immunotherapy in patients with brain metastases or with GBM.

ii. Diffusion kurtosis imaging (DKI) MRI enables quantification of non-random diffusion of water due to structural features present in tissues. This metric is more sensitive to structural abnormalities in gray matter compared to DTI 81, 82.

iii. *q*-space imaging MRI uses Fourier transformation of water diffusivity at high b values to quantify non-Gaussian mean water displacement, thus providing structural information about tissues. Theoretical calculations indicate *q*-space imaging MRI can detect tissue-restricted water diffusion in white matter with high sensitivity, although the sequences require relatively long acquisition times compared to alternative DWI methods 52, 82. Initial feasibility studies have used *q*-space imaging MRI in patients with low-grade glioma 52, while this technique has not yet been used to monitor response to immunotherapy in neuro-oncology.

iv. Neurite orientation dispersion and density index MRI differentiates water diffusion within linear cell structures (axons), free fluid (cerebral spinal fluid, ventricles), and constricted water flow in cells or extracellular spaces 53. Few studies have been published to date using this MRI technique for monitoring patients with GBM 53-55, none of which have used immunotherapy.

##### b. PWI MRI techniques

PWI MRI monitors time-intensity parameters of fluid flow through tissues of interest to assess vascularity in the tissues. Various PWI parameters have also been used to monitor response in several immunotherapy trials in neuro-oncology patients. Through these imaging techniques, progressive disease is identified by increased fluid flow due to neovascularization to support tumor growth. Early stages of immunotherapy may also show increased fluid movement due to inflammatory responses, subsequent stabilization of vascularity and permeability during continued response to therapy is anticipated to show lower perfusion values relative to progressive disease.

i. Dynamic susceptibility-weighted contrast-enhanced (DSC) MRI is the most common PWI technique in clinical practice. DSC MRI monitors contrast agent dynamics during the first pass of the contrast material through the brain to determine relative cerebral blood volume as a surrogate for microvessel density, relative cerebral blood flow, mean transit time of the contrast agent, and time to peak for contrast agent accumulation at a location of interest, which are all useful parameters for tumor grading and assessing response to chemo-radiotherapy. Confounders include artifacts from disruption of the BBB (accumulation of contrast agent) or from signal susceptibility at bone interfaces 49, 57, 83. Examples of DSC MRI to monitor response to immunotherapy are discussed below in Section III.

ii. Dynamic contrast-enhanced (DCE) MRI is a *T_1_*-weighted sequence acquired before, during, and after the injection of a Gd-based contrast agent and then fit to a two-compartment model to assess the rate of transfer from plasma to extravascular extracellular space (*K*_trans_), the extravascular extracellular volume (*v_e_*), the volume plasma (*v_p_*), and the “wash-out”, or the transfer from the extravascular extracellular space to the plasma (*k_ep_) 84. DCE MRI uses longer dynamics of contrast agent influx and efflux to determine BBB leakage or damage, extracellular volume (a surrogate for cell density), and fractional plasma volume. These parameters determine areas of high or low cell proliferation, which can correlate to tumor progression. As the initially selected time-activity curves and fitting parameters chosen by the MRI operator affect these parameters, defining widely acceptable standardized DCE MRI acquisition parameters for clinical practice remains a notable challenge in determining patient responses to neuro-oncological immunotherapy 49.

iii. Arterial spin label MRI utilizes endogenous signaling of water molecules within moving blood (compared to static parenchyma) before entering the brain to provide contrast for perfusion analyses. Compared to other techniques, arterial spin label MRI provides poor signal due to relaxation and to the low amount of “labeled” blood relative to “unlabeled” blood in the region of interest during the scan. This technique has not yet been widely explored in neuro-oncology patients treated with immunotherapy 49.

##### c. Microenvironment imaging techniques

Advanced imaging of hypoxic or acidic tumor microenvironments, which are commonly observed in GBM, is an alternative strategy for physiological imaging in neuro-oncology. BBB-permeable small compounds, such as the PET agent [^18^F]-fluoromisonidazole for imaging hypoxia, have favorable properties for imaging because they can access all CNS tissues and are selectively retained in hypoxic malignant regions 58, 59. MRI and MR spectroscopy (MRS) techniques (e.g., chemical exchange saturation transfer methods), some of which do not require administration of exogenous compounds for imaging, have been used to evaluate acidic microenvironments in GBM patients 85, 86. These physiological imaging strategies have not yet been assessed during immunotherapy treatments in neuro-oncology patients. With efficacious treatment, non-invasive imaging would be expected to indicate higher oxygenation (with less retention of hypoxia imaging agents) and greater extracellular pH in the tumor microenvironment over time.

#### 3. Molecular imaging techniques

Molecular imaging techniques visualize specific metabolites, proteins, or receptors associated with a desired cell population, tissue, or pathology, with a goal of providing diagnostic or prognostic information regarding a biological condition or process. Molecular imaging for neuro-oncology (Figure 1) is particularly attractive as it can provide information regarding spatial-temporal tumor heterogeneity and is useful for biopsy planning 44, 87. Many molecular imaging agents contain an MRI, PET, or SPECT reporter attached to a targeting component that interacts with the desired biological target after systemic administration of the imaging agent. Examples include imaging agents for amino acid PET, antibody-based PET or MRI, proliferation PET, and reporter gene/reporter probe PET or MRI. MRS is an alternative technique that uses clinical MRI scanners to monitor a characteristic molecule within a tissue of interest. This information can then be applied to diagnose or define the stage of disease in neuro-oncology. For instance, lipid and lactate signals in MRS can help distinguish lower grade tumors from higher grade tumors. Lactate, in particular is useful, as it is associated with necrotic tissue. 2-hydroxyglutarate detection by MRS can also be used to confirm the presence of an isocitrate dehydrogenase mutant tumor. The most widely used molecular imaging agent for PET in the clinic, FDG, is a marker of glucose metabolism and is transported across the BBB to freely access CNS tissues 88. The utility of FDG in neuro-oncology and immunotherapy response assessment is limited, because FDG is avidly taken up in many malignant cells, normal astrocytes, and activated inflammatory cells associated with treatment-related effects and pseudoprogression. Therefore, FDG has low specificity in identifying responses to immunotherapy. There is great interest in developing molecular imaging strategies that complement information from FDG imaging and provide specific biomarkers for response assessment in neuro-oncology 87, 89.

##### a. Amino acid PET

**Figure 2:**
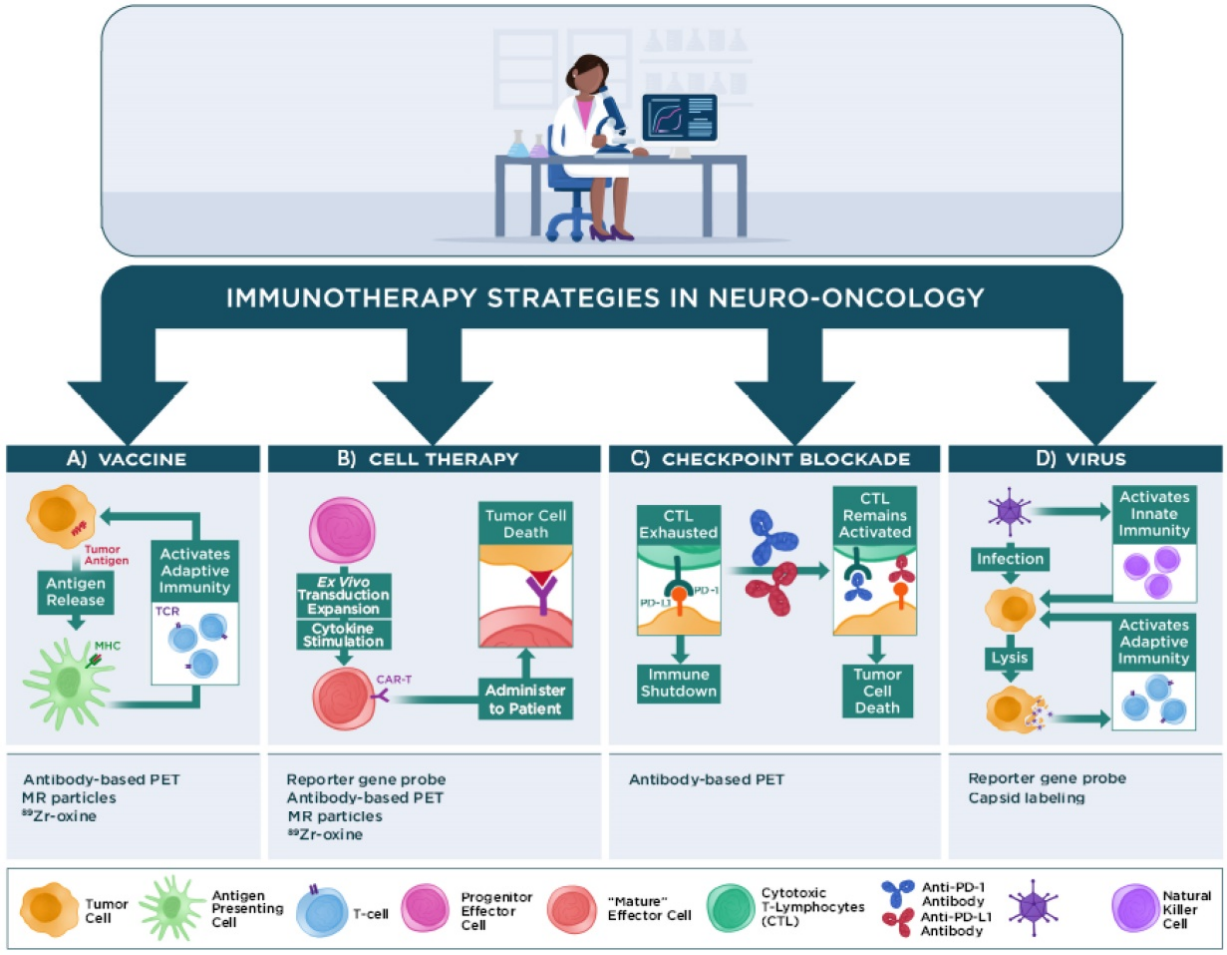
** Main classes of immunotherapy strategies used in neuro-oncology.** Vaccine immunotherapy strategies seek to load antigen presenting cells with tumor antigens for major histocompatibility complex (MHC) presentation to T cells to activate an adaptive immune response against the antigen-expressing tumor cells. Imaging the immune response can employ PET or MR reporter-labeled antibodies that specifically recognize immune cells, or immune cells loaded ex vivo with MR or PET reporters (e.g., nanoparticles, ^89^Zr-oxine) prior to injection into the subject. Cell-based immunotherapy strategies typically employ effector immune cells that are genetically modified (e.g., to express a chimeric antigen receptor [CAR]) to recognize a tumor antigen prior to re-administration into a subject. The activated cells can then proliferate and traffic to the tumor and effect tumor cell death. Imaging the immune response can employ PET or MR reporter-labeled antibodies that specifically recognize the modified immune cells, reporter gene/PET reporter probes, or cells loaded with PET or MR reporters prior to injection into the subject. Checkpoint blockade immunotherapy uses antibodies that bind to immune checkpoint proteins (e.g., programmed death-1 [PD-1]) on immune cells or the cognate proteins (e.g., programmed death-ligand 1 or 2 [PD-L1/PD-L2]) on tumor cells to prevent shutdown of cytotoxic T lymphocyte (CTL) activity by tumor cells that express these proteins. Imaging strategies can employ radiolabeled antibodies that bind to these immune checkpoint proteins. Virus immunotherapy strategies use genetically modified oncolytic or other replication-competent viruses or viral-based vectors to activate both innate immune cell (e.g., natural killer cells, macrophages) and adaptive immune cell responses against poorly immunogenic tumors. Imaging the virus can employ reporter gene/PET reporter probes, viruses with directly labeled capsids prior to injection into the subject, and other strategies used for monitoring immune responses.

Amino acid PET imaging is increasingly being recognized for its potential clinical utility in neuro-oncology 37, 38. The most common PET amino acid analogs target system L amino acid transporters and include L-[^11^C]methionine ([^11^C]MET, Figure 1E,F), *O*-(2-[^18^F]fluoro-ethyl)-L-tyrosine ([^18^F]FET), and 3,4,-dihydroxy-6-[^18^F]fluoro-l-phenylalanine ([^18^F]FDOPA). System L amino acid transporters are overexpressed in malignant glioma and CNS metastases relative to normal cells 45, 88-93. Low background uptake of amino acid analogs in normal brain provides increased contrast in brain tumors with these imaging agents. The system L family member LAT1 is also expressed on the BBB, which allows the compounds to accumulate in non-contrast enhancing tumors, although reversible transport of the compounds across cell membranes and in some cases metabolism reduce prolonged retention of the compounds within the tumor cells. Despite these limitations, amino acid PET tracers have been shown to distinguish between malignant cells and chemo-radiation treatment-related effects in neuro-oncology applications 37, 38. PET imaging with amino acid analogs would be expected to show reduced retention of the tracers in malignant CNS lesions that respond to immunotherapy due to decreasing numbers of proliferating tumor cells over time. Section III below discusses several studies that utilized amino acid PET during immunotherapy treatments in neuro-oncology patients.

##### b. Antibody-based PET or MRI

Antibodies or antibody derivatives that bind to prognostic or therapeutically relevant proteins or receptors overexpressed on tumor cells (PD-L1, EGFR, VEGF-A, etc.) or immune cells (PD-1, CTLA-4, CD8, etc.) are gaining use in oncology applications. Attaching a PET radionuclide or MRI contrast agent to these macromolecules provides a diagnostic companion for “antibody-based” molecular imaging of the target cells or tissues 62, 94, 95. These macromolecules access GBM and CNS metastases through disrupted regions of the BBB, though they have limited utility to reach CNS tissues where the BBB remains intact (e.g., low grade glioma or non-enhancing regions of GBM). Therefore, antibody-based imaging in neuro-oncology has not been investigated as extensively as small molecule PET tracers. While antibody-based imaging has been used for oncologic imaging of systemic disease, this strategy has not yet been used to determine responses to immunotherapy in clinical neuro-oncology trials. Antibody-based imaging strategies are anticipated to enable visualization of specific cell populations, such as activated immune cells, to help distinguish tumor progression from immune cell trafficking and inflammation. The kinetics or spatial distribution of the imaging agent could be used to identify novel correlates of immunotherapy response patterns over time.

##### c. PET for detection of cellular proliferation

PET compounds that indirectly monitor cell proliferation (e.g., [^18^F]fluorothymidine, [^18^F]FLT) have been used in clinical neuro-oncology studies. As normal astrocytes are not significantly proliferative, proliferation PET imaging has shown encouraging results in monitoring GBM progression in pilot human studies 96. It is not yet clear from the limited number of immunotherapy studies to date if this imaging technique is adequate to distinguish between malignant progression and inflammatory cell recruitment in neuro-oncology patients 63.

##### d. Reporter gene/reporter probe (MRI, PET, SPECT)

One molecular imaging approach is the introduction of a “reporter gene” into desired cells to express a protein, typically a kinase or extracellular receptor, not normally expressed in non-target tissues. An MRI, PET, or SPECT molecular “reporter probe” that interacts with the product of the reporter gene and specifically accumulates in the target tissue is then administered to the subject for imaging. This approach, termed reporter transgene imaging, is desirable for longitudinal imaging of cell trafficking and viability following administration of the reporter gene 64, 68, 97. A limitation for neuro-oncology applications is that the reporter molecule must be able to cross the BBB in order to detect target cells throughout CNS tissues after systemic administration. As discussed further in Section III below, this approach has been used for imaging tumor cells and immune cells in different pilot studies in neuro-oncology patients, including those treated with immunotherapy.

##### e. MRS

MRS techniques evaluate characteristic signals in the magnetic spectrum of a tissue to determine the relative amounts of specific molecules within the tissue. ^1^H (proton) spectra are the most commonly used MRS parameters, although other nuclei, including ^31^P or metal ions, have also been measured by MRS and have demonstrated potential utility to monitor malignant growth during pilot studies in neuro-oncology patients 37, 40, 86, 92. In commonly used clinical ^1^H MRS, myoinositol, choline, creatine, amino acids, *N*-acetylaspartate, and lipid/lactate picks are captured. The choline peak is a marker of cell membrane turnover, the creatine peak indicates cellular energy reserves, and *N*-acetylaspartate is for normal neurons. High tissue concentration of lipid/ lactate or specific amino acids can be measured using MRS. Classically, gliomas show a reverse of the normal ratio of choline to *N*-acetylaspartate or creatine (high creatine, low *N*-acetylaspartate) by MRS analyses when compared to normal brain parenchyma (high *N*-acetylaspartate, low creatine) due to loss of normal neurons and high cell membrane turnover associated with tumor cell proliferation. The myoinositol peak is a marker of tissue inflammation 98 but has not been explored in the setting of cancer immunotherapy. In the context of immunotherapy, tumor response would likely show an initially high creatine level that would diminish over the course of treatment while myoinositol may remain moderately high if the microenvironment was significantly inflamed by immune cell activity. In progressive disease, the creatine level relative to *N*-acetylaspartate would remain high due to continued malignant cell proliferation.

## III. Clinical immunotherapy strategies and studies incorporating advanced imaging to monitor responses to therapy

### 1. Imaging tumor-related responses during immunotherapy

Similar to the boom in immunotherapy trials for non-CNS malignancies, a growing number of pilot studies and phase I, II, or III clinical trials have tested the efficacy of various types of immunotherapy to impact patient survival in neuro-oncology. The majority of these studies are beyond the scope of this review and will not be discussed. The following sections briefly discuss four main classes of immunotherapy regimens that are under investigation in neuro-oncology and how advanced physiological or molecular imaging techniques have contributed to assessing or predicting patient responses to these treatment regimens in human patients: 1) vaccine immunotherapy, 2) cell-based immunotherapy, 3) checkpoint inhibitor immunotherapy, and 4) virus immunotherapy (Figure 2). Recent reviews are available that provide further details regarding these and other immunotherapy approaches for neuro-oncology 6, 9, 11, 99-102.

#### a. Vaccine immunotherapy and advanced imaging studies

Vaccine immunotherapies (Figure 2A) seek to stimulate immune responses initiated by presentation of tumor cell antigens in antigen-presenting cells through the extracellular major histocompatibility complex (MHC). T-cells that encounter these antigen-primed antigen-presenting cells clonally expand, circulate and encounter antigen-bearing tumor cells that they subsequently destroy directly through cytolytic mechanisms or indirectly through cytokines they produce. Vaccine immunotherapies employed in neuro-oncology include tumor cell-based, dendritic cell-based, and peptide-based vaccines, among others 99, 106, 107. Advanced imaging techniques have been most frequently employed in neuro-oncology trials with vaccine immunotherapies.

##### i. Physiological imaging of response during vaccine immunotherapy

A study of 21 pediatric patients with DIPG treated subcutaneously with a peptide vaccine after radiation therapy used DWI ADC parametric response maps to determine changes in voxel signals over time as a predictive biomarker for pseudoprogression 108. Imaging was performed during therapy and within 12 weeks of the final treatment dose. Four patients with pseudoprogression demonstrated increased fractional ADC relative to decreased fractional ADC (increased parametric response map ratio), while no difference in mean ADC was observed between pseudoprogression and true progressive disease. A longer median survival associated with treatment was observed in individuals with pseudoprogression (19.5 month) compared to those without pseudoprogression (12.5 month), although the statistical significance of this difference was not provided. A separate study of 8 patients with recurrent GBM treated with a dendritic cell vaccine after surgical resection measured PWI DSC and DWI ADC MRI parameters in brain regions showing contrast-enhancement and FLAIR signals 47. The study intended to determine differences in relative cerebral blood volume and ADC parameters at early and late time points (before and after therapeutic outcomes were known) in patients who had stable disease, those with suspected (but not confirmed) progression, and those with definitive progressive disease. The maximum relative cerebral blood volume ratios in lesions relative to normal brain were lowest in patients with stable disease and highest in those with definitive progression (Figure 1C, D), while the minimum ADC (in contrast-enhancing areas) was lowest in the pre-progression group relative to the other groups. The minimum ADC values were not as prominent compared to the relative cerebral blood volume values in defining the different response outcomes, and there was no significant difference in mean ADC between groups. An earlier study used DSC MRI to evaluate a cohort of 6 patients treated with an autologous irradiated tumor cell vaccine, as well as 2 non-treated control patients 56. All patients had been treated with surgery and radiotherapy before the study. Following vaccine therapy, relative cerebral blood volume values were generally elevated in contrast-enhancing regions, although some mismatch (low relative cerebral blood volume despite increasing contrast-enhancing area) was observed in patients during recurrence. Overall, treated patients had longer survival than non-treated patients, although relative cerebral blood volume values did not correlate with treatment or with survival time. Other studies have also used DKI DWI and DSC PWI MRI parameters to assess GBM patients treated with dendritic cell vaccines 51.

##### ii. Molecular imaging of response during vaccine immunotherapy

**Figure 3:**
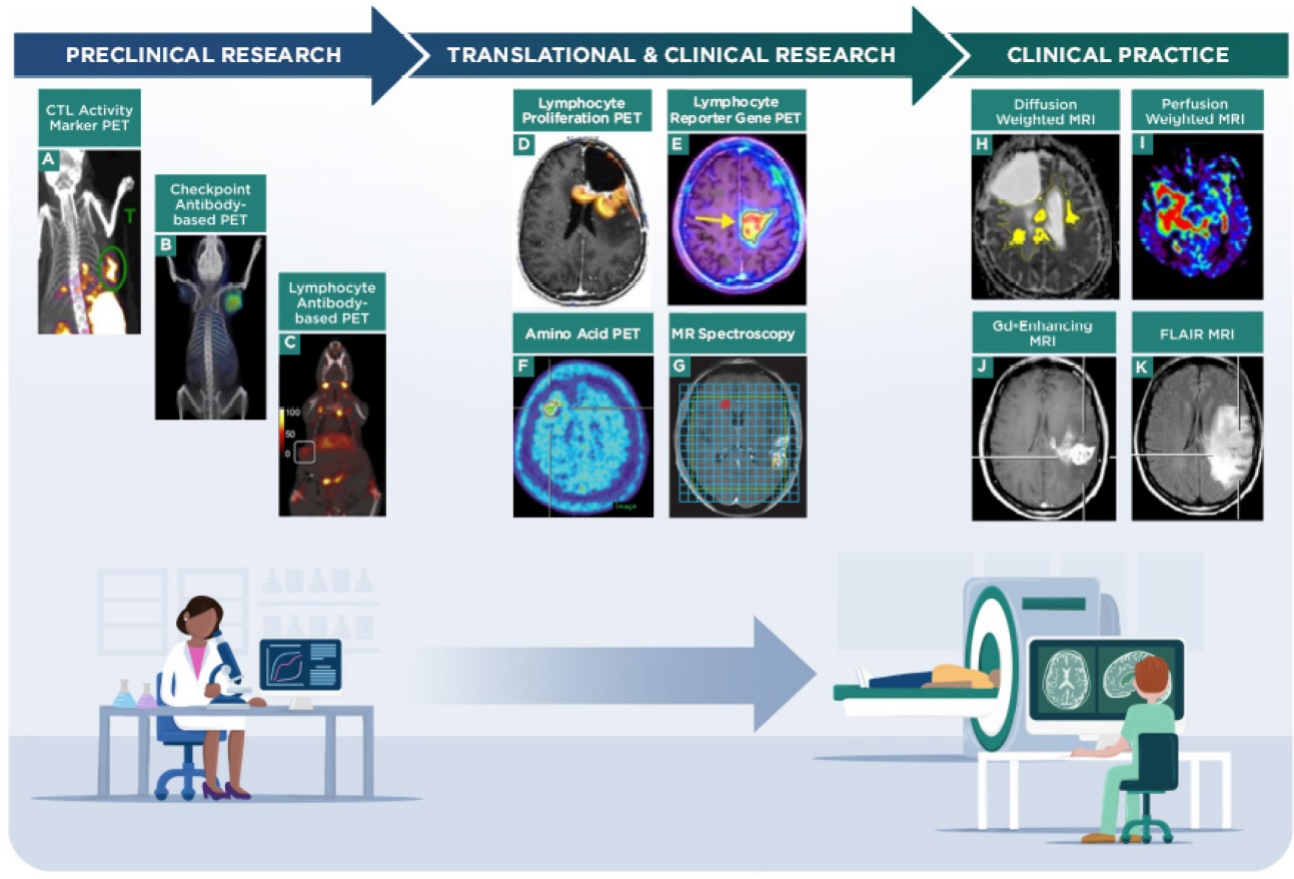
** Imaging strategies on the current research and clinical spectrum for evaluating response to immunotherapy in neuro-oncology.** Preclinical imaging strategies are being developed to distinguish immune cells from tumor cells and to correlate patterns of imaging to response to immunotherapy. **A,** granzyme B PET imaging in a murine colon carcinoma model, representing a strategy to monitor CTL activity during immunotherapy (adapted with permission from 74 copyright 2017 American Association for Cancer Research). **B,** checkpoint inhibitor antibody-based PET imaging in a Chinese hamster ovary xenograft, representing a strategy to evaluate response to checkpoint immunotherapy (adapted with permission from 103 copyright 2016 American Chemical Society). **C,** CD8 antibody fragment-based PET imaging in a murine mammary tumor model, representing a strategy to monitor lymphocyte trafficking during immunotherapy (adapted with permission from 104 copyright 2017 Rockefeller University Press). Translational and clinical research imaging strategies are being tested to determine the benefits of PET (e.g., amino acids, proliferation or transgene probes) and MR spectroscopy techniques in assessing tumor burden and response to immunotherapy treatments. **D,** 2-chloro-2'-deoxy-2'[^18^F]fluoro-9-β-D-arabinofuranosyl-adenine PET imaging of a GBM patient treated with vaccine and checkpoint inhibitor immunotherapy, to monitor lymphocyte proliferation and trafficking in the tumor (adapted with permission from 67 copyright 2017 Antonios JP et al.). **E,** 9-[4-[^18^F]fluoro-3-(hydroxymethyl)butyl]guanine PET imaging via a reporter gene in a GBM patient treated with CAR-T cells, to monitor lymphocyte viability and trafficking in the tumor (adapted with permission from 68 copyright 2017 American Association for the Advancement of Science). **F,** [^18^F]FET amino acid PET imaging of a CNS lesion in a metastatic melanoma patient treated with checkpoint immunotherapy, to monitor tumor response to treatment (adapted with permission from 60 copyright 2016 Oxford University Press). **G,** MRS of a GBM patient treated with dendritic cell immunotherapy, to monitor tumor response to treatment (adapted with permission from 49 copyright 2017 Aquino D et al.). Clinical practice uses established MR techniques (e.g., contrast-enhancement, diffusion-/perfusion-weighted imaging, FLAIR sequences) to evaluate tumor burden and are currently used to refine and identify novel quantitative metrics that correlate with patient outcomes. **H,** diffusion weighted MRI showing ADC volumes in a GBM patient treated with checkpoint immunotherapy (adapted with permission from 105 copyright 2017 Springer-Verlag Berlin Heidelberg). **I,** perfusion weighted MRI showing relative cerebral blood flow in a GBM patient (adapted with permission from 83 copyright 2015 Springer Nature). Representative Gd-contrast **(J)** and FLAIR MRI **(K)** images in a GBM patient to indicate structural MRI assessment (adapted with permission from 15 copyright 2017 Informa UK Limited).

A PET molecular imaging study used [^11^C]MET to define parametric response maps in 14 patients with recurrent glioma treated with WTI peptide immunotherapy (Figure 1E,F) 48. Voxel-wise PET analysis was performed in MRI contrast-enhancing areas of the tumor in the study. The data indicated that using a 5% threshold for the percent increase of [^11^C]MET area differentiated non-responders from responders. An early study reported results of MRS analyses in two patients with recurrent GBM patients after surgery, radiation therapy, and IL4-toxin conjugate immunotherapy in conjunction with temozolomide chemotherapy 69. Conventional Gd-*T_1_* MRI showed contrast enhancement in the lesions while choline MRS showed minimal signal in the tumor in this study, indicating pseudoprogression. Following treatment, however, both patients had rapid recurence of disease indicated by an increase in choline MRS signals, conventional imaging, and clinical decline. While this study provided simplified MRS analyses in only small regions of interest, the results support future work with MRS to predict response to combinatorial treatment regimens that incorporate immunotherapy in neuro-oncology (Figure 3).

#### b. Cell-based immunotherapy and advanced imaging studies

In cell-based immunotherapy strategies (Figure 2B), precursor cells from the innate or adaptive immune system are collected from patients or healthy donors, transformed or activated *ex vivo*, and infused into the patient as effector immune cells that recognize and destroy specific types of antigen-presenting tumor cells without requiring antigen-presenting cell-mediated priming. Classes of cell- based therapies in neuro-oncology include chimeric antigen receptor (CAR) T-cells (and their variants), lymphokine-activated killer cells, T-cell receptor-transduced T-cells, and activated tumor infiltrating lymphocytes, among others 101, 109, 110.

Advanced imaging has also been used to monitor tumor responses during cell-based immunotherapy 67, 68. As discussed further below, several of these studies focused primarily on imaging the immune cell response and correlating those parameters to changes in tumor growth as estimated by supporting imaging techniques. It is not yet clear which advanced imaging parameters are suitable to distinguish between tumor progression and immune cell recruitment at early stages of treatment.

#### c. Checkpoint inhibitor immunotherapy and advanced imaging studies

Checkpoint inhibitor immunotherapies (Figure 2C) were designed to overcome immunosuppressive cell-cell signaling mechanisms between malignant cells and effector immune cells. Monoclonal antibodies (mAbs) that bind to cytotoxic T lymphocyte-associated protein 4 (CTLA-4), programmed death-1 (PD-1), or programmed death-ligand 1 (PD-L1) have been successful at mediating immunotherapy responses in subgroups of patients with melanoma, non-small-cell lung cancer, or renal cell carcinoma, among others 8, 10. The only completed randomized phase III trial to date evaluating the outcome of PD-1 checkpoint immunotherapy (nivolumab) on GBM patient survival showed no benefit from treatment with nivolumab relative to treatment with bevacizumab 111. Ongoing clinical trials are evaluating the efficacy of other checkpoint inhibitor immunotherapies and combination treatments in neuro-oncology patients 102, 112-114. Several pilot studies have sought to find correlations between various advanced imaging parameters and therapeutic outcomes in neuro-oncology patients treated with checkpoint inhibitor immunotherapy.

##### i. Physiological imaging of response during checkpoint inhibitor immunotherapy

A study of 10 patients with recurrent GBM treated with anti-PD-1 and/or anti-CTLA-4 checkpoint inhibitor immunotherapy used intermediate ADC DWI volume changes within FLAIR regions of relatively high cellularity to correlate imaging parameters with response to immunotherapy (Figure 3) 105. The imaging findings of lower ADC volumes at later time points predicted therapeutic response in all 10 patients, despite initial increases in ADC volumes. Patients deriving more therapeutic benefit from therapy were identified by ADC parameters at a median time of 93 days after treatment while conventional MRI was not conclusive until a median time of 121 days after treatment (the statistical significance of this comparison was not reported). The authors determined that therapeutic outcome (time to progression) was better correlated with the ADC volume than with 2-dimensional RANO measurements, FLAIR, or Gd-contrast enhancement. The study noted that careful selection of intermediate ADC values is required for correct assignment of therapeutic outcome and indicated that future studies would be needed to demonstrate the widespread utility of this parameter. While diffusion weighted imaging identified therapeutic response earlier than conventional MRI in this patient cohort, it is desirable to find parameters that define response at even earlier time points (e.g., 1-2 months) after initiating checkpoint immunotherapy so non-responding patients could be promptly switched to an alternative therapeutic regimen.

##### ii. Molecular imaging of response during checkpoint inhibitor immunotherapy

A retrospective study assessed five patients with melanoma brain metastases who were given ipilimumab or nivolumab prior to PET imaging with [^18^F]FET (Figure 3) 60. From this cohort of patients, one who was treated with ipilimumab demonstrated pseudoprogression while all others had progressive disease. The tumor-to-background and time to peak PET signals were higher and faster, respectively, in patients with progressive disease compared to the patient that showed pseudoprogresion. Pseudoprogression by conventional MRI has been observed during checkpoint inhibitor immunotherapy in many case reports for brain metastases of non-small cell lung cancer 115, melanoma (Figure 1A, B) 46, and other malignancies 116. In these cases, histopathology confirmed pseudoprogression; alternative imaging analyses were not reported. These results indicate an opportunity for additional prospective studies utilizing advanced imaging approaches to help define prognostic outcomes for patients with CNS metastases. The growing use of checkpoint inhibitors has prompted development of companion diagnostic imaging agents to quantify target expression and aid response assessment in preclinical (Figure 3) and early phase clinical studies 62, 117- 120. A first-in-human study recently demonstrated that ^89^Zr-nivolumab and the anti-PD-L1 adnectin ^18^F-BMS-986192 accumulated in lesions in 13 patients with advanced non-small cell lung cancer, where uptake of the radiolabeled agents correlated with histological staining and subsequent response to immunotherapy with nivolumab 121. Low uptake of ^89^Zr-nivolumab and ^18^F-BMS-986192 was apparent in brain metastases in two patients from the study, although not all of the brain metastases showed accumulation of the PET signals. These preliminary findings will need to be validated through ongoing and future studies to determine if macromolecule- based PET imaging of checkpoint protein expression is a viable strategy to predict response to checkpoint inhibitor immunotherapy in patients with primary or metastatic CNS lesions.

#### d. Virus immunotherapy and advanced imaging studies

Virus immunotherapy strategies (Figure 2D) use live, immunogenic viruses that replicate in tumor cells to stimulate innate and adaptive immune responses within the tumor environment. Oncolytic viruses selectively replicate in tumor cells rather than normal cells and directly lyse the host tumor cells, releasing additional tumor antigens that further propagate the immune response. Oncolytic virus immunotherapy can also change the tumor microenvironment to favor an antitumor response by reprogramming the polarization of tumor-associated macrophages 122. Several viral immunotherapy strategies are currently in clinical evaluation in neuro-oncology patients or various stages of translational development, as previously reviewed 123-129.

##### i. Molecular imaging of response during virus immunotherapy

PET or SPECT probes have been used in GBM patients to monitor tumors infused with oncolytic virus and viral-mediated gene therapy via the *HSV-tk* reporter gene. PET imaging showed intra-tumor accumulation of the reporter probe in one of four patients treated with an adenovirus and chemotherapy 64, while SPECT imaging did not show accumulation of the reporter probe in tumors from eight patients treated with an oncolytic herpes simplex virus 65. These studies indicated that viral infection rates, the ability of the imaging probe to cross the BBB, lysis of infected tumor cells, and the sensitivity of the imaging technique can influence the ability of molecular imaging to monitor viral replication in GBM patients. It is not yet clear if these techniques to monitor viral replication can be applied to predicting response to virus immunotherapy in clinical neuro-oncology applications.

#### e. Other advanced imaging studies during immunotherapy

A pilot study used [^18^F]FLT PET and conventional MRI to assess five individuals with melanoma brain metastases, three of whom were treated with various types of immunotherapy after failing prior chemotherapies 63. All patients showed high uptake of [^18^F]FLT in the tumor (median standardized uptake value ratio of 9.9 relative to background) at initial assessment. Only one of the five patients had both pre- and post-immunotherapy assessment by [^18^F]FLT, so no definitive conclusions could be made regarding changes in PET signal and therapeutic outcome. This patient was evaluated by PET with [^18^F]FLT upon entrance to the study and one month after treatment with nivolumab and ipilimumab. The second PET/MRI scan showed a mixed partial response in the five evaluable CNS lesions, where three lesions decreased in size and showed lower uptake of [^18^F]FLT relative to the baseline image. New, small lesions (less than 1 cm diameter) were also observed on the post-immunotherapy MRI scan, although these lesions did not show appreciable accumulation of [^18^F]FLT. The patient was switched to an alternative therapy and not further evaluated by PET in the reported study. The results from this study warrant future work to determine correlations between [^18^F]FLT signals in the tumor and outcomes to immunotherapy in neuro-oncology patients.

### 2. Imaging immune cell-related responses during immunotherapy

Combining molecular imaging of immune or inflammatory cells with morphologic assessment is another approach to determine response to immunotherapy in neuro-oncology 61, 101, 130, 131. This approach could help identify individuals demonstrating immune cell activation or trafficking in the tumor and who would be candidates to remain on immunotherapy treatment if morphologic imaging or other analyses (e.g., biopsy) do not conclusively define progressive disease. The scope of the discussion below is restricted to human neuro-oncology studies that utilized advanced imaging to evaluate response to immunotherapy.

a. PET, MRI, or SPECT immune cell tracking. A common technique is to pre-load or label cells *ex vivo* with a contrast agent for MRI (e.g., ferumoxytol), PET (e.g., ^89^Zr-oxine), or SPECT (e.g., ^111^In-oxine) imaging, inject the labeled cells into the patient, and monitor the signals to determine the trafficking and duration of cell localization in the target tumor region 70, 71, 97, 132. While SPECT and MRI imaging with pre-labeled immune cells has been widely used in clinical practice for other applications, the pre-label cell imaging strategy has not yet been clinically validated in neuro-oncology patients treated with immunotherapy. Challenges with the technique include the inability to distinguish between signals from viable cells and dead cells, as well as dilution of contrast (resulting in low signal) in viable cells over time as the cells divide and traffic throughout the patient.

b. Transgene/reporter gene studies with immune cells. Imaging of transgene expression through a PET reporter has been used in clinical research to monitor immune cell trafficking and response to therapy. An initial case report published in 2009 66 used CD8^+^ CTLs that had been genetically modified to express chimeric antigen receptor (CAR) interleukin-13 receptor alpha 2 for tumor targeting as well as herpes simplex virus type 1 thymidine kinase for dual suicide gene therapy and PET imaging of the CAR cells using 9-[4-[^18^F]fluoro-3-(hydroxymethyl)butyl]guanine. A recent publication demonstrated the results of this strategy in six additional patients with recurrent GBM (Figure 3) 68. The uptake of 9-[4-[^18^F]fluoro-3-(hydroxymethyl)butyl]guanine increased in all brain lesions after injection of the transformed CAR cells relative to the pre-treatment PET scan with the tracer. The pattern of the increase in the PET signal intensity and volume of distribution varied among patients, thus precluding the definitive assessment of CAR cell trafficking to the tumors in the small cohort of patients. These studies highlight the importance of distinguishing between non-specific pooling of PET imaging agents and specific immune cell trafficking within GBM tumors to assess immunotherapeutic efficacy through transgene PET imaging.

c. A similar approach to the transgene/reporter molecule imaging strategy above is to use a reporter molecule that becomes enzymatically trapped in target immune cells without the need for prior genetic manipulation of the cells. In a translational research study, three GBM patients were given 2-chloro-2'-deoxy-2'[^18^F]fluoro-9-β-D-arabinofuranosyl-adenine and imaged by PET prior to and following treatment with a dendritic cell vaccine in combination with pembrolizumab or bevacizumab (Figure 3) 67. The reporter molecule in this strategy becomes trapped in immune cells that endogenously express a kinase not expressed in most normal tissues. Patients were also imaged by DWI and PWI MRI to determine pre- and post-treatment changes in ADC and cerebral blood volume parameters. Comparing the pre- and post-treatment scans enabled determination of an index that measured immunotherapy response relative to tumor response. Preclinical models of GBM showed that the immunotherapy response index determined through PET imaging correlated with response to therapy. These initial results support future studies in larger cohorts of patients with GBM to determine if PET imaging with 2-chloro-2'-deoxy-2'[^18^F]fluoro-9-β-D-arabinofuranosyl-adenine is a valid approach to predict response to immunotherapy regimens.

The above studies demonstrate both opportunities and challenges associated with imaging responses to immunotherapy in neuro-oncology. Ongoing research is needed to define how biomarker expression or other imaging metrics (e.g., kinetic changes in tumor growth at early time points after initiating therapy) correlate with clinical outcomes during therapy. Future trials incorporating larger cohorts of patients and their long-term outcomes to therapy will be needed to discern the most reliable metrics for monitoring responses to immunotherapy regimens.

## IV. Groundbreaking preclinical imaging approaches and alternative methods to detect immune responses during immunotherapy for future assessment in neuro-oncology

Besides the approaches discussed above, additional imaging agents and analytical techniques are being developed to define prognostic parameters for immunotherapy responses 61, 95, 97, 110, 130-134, many of which have not yet been attempted in neuro-oncology. Several antibody fragment-derived PET probes that target immune cell lineage proteins (e.g., CD3, CD4, CD8) have demonstrated correlations between immune cell trafficking and tumor response to checkpoint inhibitor immunotherapy in syngeneic animal models of cancer (Figure 3) 72, 73, 104, 135. Strategies to detect immune cells through analogous humanized PET probes are now in the early stages of clinical development. Alternative molecular imaging techniques that specifically detect cytolytic activity of effector immune cells (e.g., CTLs) have been shown to predict therapeutic outcomes in preclinical models of non-CNS malignancies 74. PET imaging of a modified human T-cell receptor transduced in T cells in preclinical cancer models correlated with the number of infiltrating T cells, supporting the concept for a generalizable strategy to precisely detect activated CTLs during cell-based immunotherapy (Figure 3) 75. Exploring these strategies in neuro-oncology models would provide valuable data to complement existing techniques that detect immune cell trafficking or tumor cell responses. Future work would be required to determine if these novel, advanced imaging approaches provide correlates between imaging and therapeutic outcomes that can positively impact patient care. As reviewed elsewhere, flow cytometry analyses of immune cells in peripheral blood and additional approaches besides non-invasive imaging are being explored to define prognostic parameters for immunotherapy in translational and clinical neuro-oncology research 136, 137.

## V. Conclusions and future outlook

Expansions in advanced imaging have catalyzed the emergence of research and clinical strategies defining patterns of response to immunotherapy for neuro-oncology patients. However, significant obstacles must be overcome prior to routine clinical adoption of experimental imaging strategies. As the incidence of GBM is low and patients with secondary CNS metastases often have confounding clinical factors (e.g., systemic disease), a main hurdle has been implementing prospective clinical trials with sufficient numbers of neuro-oncology patients to demonstrate statistically reliable correlates between imaging parameters and therapeutic outcomes during immunotherapy. Most patients in these trials have received multiple, varying courses of therapy before enrollment in immunotherapy trials, further complicating the assignment of defined imaging response patterns to a specific therapeutic regimen. An additional challenge is in implementing standardized terminology and response criteria across neuro-oncology studies, as a recent report found that 63% of neuro-oncology publications after 2010 did not use the RANO criteria 138. Greater uniformity in implementing response metrics in current and future studies would aid in comparing the relative efficacy of novel immunotherapy regimens in neuro-oncology trials.

Most advanced imaging approaches to evaluate response to immunotherapy are still in initial, exploratory stages in neuro-oncology research. For the field to make rapid, meaningful advancement, it will be important to reach consensus across studies and institutions regarding acquisition parameters and reported findings for a particular imaging technique 62. Many exploratory imaging studies to date have utilized varied, specialized instrument parameters during image acquisition, particularly for advanced MRI applications, that have hampered comparison of imaging results across studies 139. Consensus recommendations exist for several advanced imaging techniques for neuro-oncology or other brain imaging applications 39, 140-142. There are currently no established, standardized guidelines in neuro-oncology to compare advanced imaging techniques or parameters in routine clinical practice or in clinical trial design. However, groups such as the Quantitative Imaging Network with the National Cancer Institute and the Quantitative Imaging Biomarkers Alliance with the Radiological Society of North America are currently working on formulating such recommendations. Criteria that define tumor response or progression through quantitative PET analyses using [^18^F]-FDG are available from the European Organization for Research and Treatment of Cancer (EORTC) for glioma and brain metastases, and from PET Response Criteria in Solid Tumors (PERCIST) guidelines for brain metastases. While recommended guidelines to compare multi-modality imaging parameters have not yet been established, integrating imaging parameters from various modalities has been shown in previous studies to increase diagnostic specificity and sensitivity relative to separate, single-modality imaging in neuro-oncology patients 139, 143. Consensus recommendations that outline schema to integrate imaging methods and biomarkers into research and routine practice have been proposed by the EORTC 144. Utilizing standardized acquisition parameters and assessment criteria across relevant studies will most effectively enable the research and clinical communities to determine whether or not these parameters can be effectively used to predict therapeutic responses in neuro-oncology patients.

Imaging scientists should work closely with referring physicians, neuro-oncologists, and the clinical research team to best integrate advanced imaging into trial design and patient care. Considering the limited patient population and the poor prognosis associated with primary or metastatic CNS malignancies, simultaneously enrolling the same patients into both immunotherapy and imaging trials would enable streamlined evaluation of non-invasive imaging methods and responses to immunotherapy. Incorporating advanced imaging sessions into large cohorts of patients in phase II or III immunotherapy trials would aid accumulation of sufficient patient data to yield statistically meaningful results for both therapeutic outcome and matched imaging parameters.

As addressed above, distinguishing tumor progression from inflammation and other treatment-related effects is a significant clinical challenge in neuro-oncology. Utilizing multi-modality imaging to differentiate between these separate biological processes is an attractive strategy to address this challenge. One way to potentially identify responses to immunotherapy regimens is to specifically monitor trafficking and activity of effector immune cells (e.g., CTLs 132) and compare these image findings to alternative imaging approaches that identify proliferating tumor cells (e.g., amino acid PET). Imaging agents based on antibodies or antibody derivatives that target populations of effector immune cells (e.g., CD8 imaging agents) are currently being evaluated in clinical trials 145 and are anticipated to be used in neuro-oncology patients treated with immunotherapy in the near future. Similarly, imaging strategies utilizing radiolabeled reporting molecules (particularly BBB-permeable small molecules) that selectively accumulate in either transgenic or endogenous immune cells are worthy of continued evaluation in neuro-oncology immunotherapy studies. Such imaging strategies that specifically accumulate in immune cells rather than tumor cells are anticipated to identify patients with activated immune responses within the tumor microenvironment, which is required for immunotherapy-mediated tumor regression. If patients do not demonstrate activated immune responses during a predetermined time point following treatment, they can be switched to alternative immunotherapies or other regimens as quickly as possible.

Continued development of companion diagnostic imaging agents for molecular targeted immunotherapy strategies, such as checkpoint inhibitor therapy or CAR-T cell therapy, is important for evaluating therapeutic efficacy in neuro-oncology patients. Molecular imaging strategies can help identify the presence or absence of the corresponding therapeutic target and provide rationale to initiate, continue, or terminate a particular course of therapy in individual patients 62. Strategies utilizing BBB-permeable imaging agents are advisable in neuro-oncology studies as these strategies can be used for evaluating response in enhancing and non-enhancing CNS regions, including areas with tumor cell dissemination not identifiable by conventional imaging. Ongoing and future immunotherapy research in neuro-oncology should not exclude macromolecule-based imaging agents (e.g., antibodies, peptides), as it is possible that useful prognostic information could be gained from imaging patterns in regions of BBB disruption (e.g., from kinetic or contrast maps with Gd-based agents, antibody-based PET, etc.). Imaging with BBB-impermeable agents is particularly relevant for antibody-based immunotherapies that bind to tumor cells (e.g., PD-L1 checkpoint immunotherapy), since such imaging agents accumulate in regions of the CNS where the therapeutic agents can also access and exert their primary effects.

Radiomics is an emerging field that uses automated computational algorithms to identify changes in imaging features (e.g., pixel intensity, volumetric data, etc.) across serial imaging time points, and to correlate these changes to a biological outcome (e.g., survival, tumor progression) 146. Harnessing single- or multi-modality radiomics in neuro-oncology studies, particularly those that evaluate response to immunotherapy regimens, could address the current bottleneck of manual image analyses and spark discovery of novel imaging parameters to predict outcomes of therapy. Additionally, quantitative imaging metrics related directly to biology provides biological assessment of the tumor noninvasively. The increasing availability of imaging data and speed of computation are expected to enable greater use of radiomics and quantitative imaging in the near future 147.

While much progress has been made in the fields of advanced molecular imaging and immunotherapy during the last decade, further developments are needed to determine how they can best be merged to improve the outcomes for neuro-oncology patients. No single agent or imaging technique has yet been clinically validated as a satisfactory prognostic indicator for response to immunotherapy regimens. The clinical and imaging communities eagerly await emerging results from ongoing neuro-oncology clinical trials that utilize investigational immunotherapy regimens, novel imaging strategies, and improved response criteria. As these forthcoming data become available, it is anticipated that prognostic patterns will be realized and incorporated into future practice to improve the outcomes of neuro-oncology patients.

## Figures and Tables

**Table 1:** List of non-invasive clinical and preclinical imaging techniques for monitoring response to immunotherapy in neuro-oncology studies.

CLINICAL IMAGING TECHNIQUES					
Category of imaging technique	Imaging modality	Research or Clinical Practice	Disease in neuro-oncology studies	Immunotherapy category used in neuro-oncology studies	References
Structural	Contrast-enhancing MRI	Clinical practice	GBM, CNS metastases	Vaccine, Cell therapy, Checkpoint blockade, Virus	14, 49
	FLAIR MRI	Clinical practice	GBM, CNS metastases	Vaccine, Cell therapy, Checkpoint blockade, Virus	14, 46, 49
Physiological	DTI MRI	Clinical practice	GBM, CNS metastases	Vaccine, Cell therapy, Checkpoint blockade, Virus	50
	Diffusion kurtosis imaging MRI	Research	GBM	DC vaccine	51
	*q*-space imaging MRI	Research	Glioma (low grade)	n/a	52
	Neurite orientation dispersion and density index MRI	Research	GBM	n/a	53-55
	DSC MRI	Clinical practice	GBM, CNS metastases	Vaccine, Cell therapy, Checkpoint blockade, Virus	47, 51, 56, 57
	DCE MRI	Clinical practice	GBM, CNS metastases	Vaccine, Cell therapy, Checkpoint blockade, Virus	49
	Arterial spin label MRI	Research	GBM	n/a	49
	PET hypoxia	Research	GBM	n/a	58, 59
Molecular	Amino acid PET	Research	GBM, CNS metastases	Vaccine, Checkpoint blockade	48, 60
	Antibody-based PET	Research	GBM, CNS metastases	n/a	61, 62
	Proliferation PET	Research	GBM, CNS metastases	Vaccine, Checkpoint blockade	63
	PET reporter gene	Research	GBM	Vaccine, Cell therapy, Virus	64-68
	MRS	Research	GBM	Cell therapy	69
Immune cell tracking	PET, MRI, SPECT	Research/Clinical practice	GBM	Vaccine, Cell therapy	70, 71
**PRECLINICAL IMAGING TECHNIQUES**					
**Category of imaging technique**	**Imaging modality**	**Imaging target**	**Disease model**	**Immunotherapy category used in studies**	**References**
Molecular	Antibody-based PET	Effector immune cells proteins	Syngeneic murine colon or mammary carcinoma	Checkpoint blockade	72, 73
	Peptide-based PET	Immune cell cytolytic activity	Syngeneic murine colon carcinoma	Checkpoint blockade	74
	Reporter gene PET	Transgenic T cells	Murine acute myeloid sarcoma	Cell therapy	75
	Reporter gene MRS	Virus-transfected cells	Orthotopic glioma (rat)	Virus	76

## Abbreviations

ADC: apparent diffusion coefficient in diffusion MRI
BBB: blood-brain barrier
CAR: chimeric antigen receptor
CNS: central nervous system
CT: computed tomography
CTL: cytotoxic T lymphocyte
CTLA-4: cytotoxic T lymphocyte-associated protein 4
DCE: dynamic contrast-enhanced
DKI: diffusion kurtosis imaging
DSC: dynamic susceptibility-weighted contrast-enhanced
DTI: diffusion tensor imaging
DWI: diffusion weighted imaging
FDG: 2-deoxy-2 [^18^F]fluoro-D-glucose
FDOPA: 3,4,-dihydroxy-6- fluoro-L-phenylalanine
FET: *O*-(2-fluoro-ethyl)-L- tyrosine
FLAIR: fluid-attenuated inversion recovery
FLT: fluorothymidine
GBM: glioblastoma multiforme
iRANO: immunotherapy RANO
*K*_trans_: rate of transfer from plasma to extravascular space in perfusion MRI
MET: L-methionine
MRI: magnetic resonance imaging
MRS: magnetic resonance spectroscopy
PD-1: programmed death-1
PD-L1: programmed death-ligand 1
PET: positron emission tomography
PWI: perfusion weighted imaging
RANO: response assessment in neuro-oncology
RANO-BM: RANO brain metastases
RANO-HGG: RANO high grade glioma
RECIST: response evaluation criteria in solid tumors
SPECT: single photon emission computed tomography
